# The inner ear immune microenvironment in sensorineural hearing loss: mechanisms and therapeutic perspectives

**DOI:** 10.3389/fimmu.2026.1911450

**Published:** 2026-09-09

**Authors:** Yingyuan Guo, Jingmao Lv, Dejun Zhang

**Affiliations:** 1 Department of Otorhinolaryngology, Second Hospital of Jilin University, Jilin, China; 2 Department of Spinal Surgery, Jilin Provincial People’s Hospital, Jilin, China

**Keywords:** biologics, blood-labyrinth barrier, cochlea, immune microenvironment, inflammaging, intratympanic therapy, macrophage, NLRP3 inflammasome

## Abstract

Sensorineural hearing loss (SNHL) affects over 1.5 billion individuals worldwide and remains the most prevalent sensory deficit in humans. Although classical pathological models have emphasized hair cell death from oxidative stress and mitochondrial dysfunction, accumulating evidence over the past decade has redefined the cochlea as an immunologically active tissue rather than an immune-privileged organ. Resident macrophages populate the spiral ligament, stria vascularis, spiral ganglion, and modiolus, and they orchestrate both protective and damaging responses to acoustic, ototoxic, metabolic, and immunological stress. Recent single-cell transcriptomic data have identified disease-specific macrophage subsets, including a proinflammatory CD74+CD14+ population enriched in age-related and noise-induced hearing loss. Molecular drivers of cochlear inflammation include cytokines such as TNF-alpha, IL-1beta, and IL-6, danger-associated molecular patterns including HMGB1 and HSP70, NLRP3 inflammasome activation, complement signaling, and disruption of the blood-labyrinth barrier. These mechanisms converge across diverse SNHL etiologies, from noise-induced synaptopathy and inflammaging in presbycusis, to ototoxicity, autoimmune inner ear disease, sudden idiopathic SNHL, and viral or post-viral hearing loss. Therapeutically, the field is shifting from broad-spectrum corticosteroids toward immune-targeted strategies, including cytokine-directed biologics, NLRP3 inhibitors, complement-targeted agents, macrophage reprogramming, mesenchymal stem cell-derived exosomes, intratympanic nanoparticle delivery systems, and gene therapy approaches with regulated immune profiles. Perilymph sampling now offers a window into the cochlear microenvironment in living patients, enabling biomarker-guided precision medicine. This review synthesizes the cellular, molecular, and clinical evidence supporting the cochlear immune microenvironment as a unifying axis in SNHL pathogenesis, and outlines therapeutic perspectives that may transform the standard of care over the next decade.

## 1 Introduction

Hearing loss is a leading global cause of years lived with disability and affects an estimated 1.57 billion people, with moderate-to-complete hearing loss disproportionately concentrated in regions with limited access to audiological care. Sensorineural hearing loss (SNHL), arising from damage to the cochlea, auditory nerve, or central auditory pathways, accounts for the majority of these cases and is projected to affect 2.45 billion individuals by 2050 if current trends continue (1).

Conventional pathophysiological frameworks have emphasized hair cell death driven by oxidative stress, mitochondrial dysfunction, and excitotoxicity (2). While these mechanisms remain central, they do not fully explain the temporal evolution, regional specificity, or interindividual variability observed across clinical SNHL phenotypes. Over the past decade, an expanding body of work has refocused attention on the cochlear immune microenvironment as a shared and modifiable substrate of injury across multiple etiologies (3, 4).

The inner ear was historically considered immune-privileged because of the blood-labyrinth barrier and minimal lymphatic drainage. This view has been overturned by the demonstration of a steady-state resident macrophage population within several cochlear compartments and by direct evidence of immune cell infiltration after acoustic, ototoxic, infectious, and aging-related insults (5, 6). The cochlea now appears more analogous to other partially immune-privileged tissues, such as the central nervous system, in which resident myeloid cells maintain homeostasis while contributing to disease when dysregulated.

Therapeutically, the standard of care for many SNHL etiologies, including sudden idiopathic SNHL and autoimmune inner ear disease, still relies on systemic and intratympanic corticosteroids whose benefit is modest and inconsistent (7, 8). The mechanistic rationale for steroids, namely the suppression of cochlear inflammation, paradoxically supports the relevance of immune mechanisms but also points to the inadequacy of broad immunosuppression. Precision immunomodulation, informed by the cellular and molecular architecture of the cochlear immune microenvironment, represents the most promising avenue for the next generation of therapies.

This review summarizes current understanding of the inner ear immune microenvironment in SNHL. We first describe how immune privilege has been redefined and outline the cellular and molecular components of cochlear immunity. We then synthesize the mechanistic evidence linking immune dysregulation to specific SNHL etiologies, including noise-induced, age-related, ototoxic, autoimmune, sudden, viral, and radiation-related hearing loss. Finally, we discuss emerging therapeutic perspectives that target cochlear inflammation, including biologics, inflammasome inhibitors, cell-based therapies, drug delivery technologies, and perilymph-guided precision medicine.

## 2 Redefining the cochlear immune microenvironment

The cochlea is a fluid-filled spiral organ in the temporal bone subdivided into three scalae and several anatomically distinct compartments, including the organ of Corti, stria vascularis, spiral ligament, spiral ganglion, and modiolus. Each compartment harbors a unique cellular environment with specific functional demands. The blood-labyrinth barrier (BLB) separates the systemic circulation from the cochlear fluid spaces and is essential for maintaining the endocochlear potential and ionic homeostasis required for mechanotransduction (9, 10).

The BLB is not a single barrier but a structurally heterogeneous interface, particularly within the stria vascularis. There, capillary endothelial cells are ensheathed by pericytes embedded in a shared basement membrane, while perivascular-resident macrophage-like melanocytes lie outside the basement membrane and extend processes that contact multiple capillaries (9, 10). These perivascular cells regulate tight junction integrity, immune surveillance, and local cytokine release. Disruption of the BLB increases its permeability, alters endocochlear potential, and permits trafficking of immune cells and inflammatory mediators into otherwise protected niches (11).

Historical conceptions of cochlear immune privilege rested on three observations: the absence of conventional lymphatic vessels in the cochlea, the existence of the BLB, and the limited inflammatory response observed under steady-state conditions. Subsequent work has substantially revised this picture. Immunohistochemical and three-dimensional imaging analyses have demonstrated abundant resident macrophages in the spiral ligament, spiral ganglion, modiolus, and on the scala tympani aspect of the basilar membrane, with relatively fewer within the organ of Corti itself (3, 12). Human cochlear tissues additionally contain scattered CD4+ and CD8+ T lymphocytes, particularly in the modiolus and adjacent to vessels, suggesting that adaptive immune surveillance occurs even in the absence of overt disease (6).

Recent single-cell transcriptomic studies in mouse models have provided a more nuanced view of cochlear immune heterogeneity. In age-related hearing loss (ARHL) and noise-induced hearing loss (NIHL), a proinflammatory CD74+CD14+ macrophage subset becomes expanded, with enhanced cellular communication with spiral ganglion neurons (13). In aged stria vascularis, basal cells overexpress CX3CL1 while macrophages upregulate CX3CR1 and TNF-alpha, identifying the fractalkine axis as a potential trigger of the local inflammatory state in presbycusis (14). These data argue that the cochlear immune compartment is not a uniform population but a network of regionally specialized cell types whose activity is shaped by local cues and systemic factors.

The location of immune cells is functionally significant. Macrophages adjacent to the inner hair cell synaptic region appear to support synaptic maintenance and repair after acoustic injury, whereas macrophages activated within the spiral ligament or stria vascularis can promote tissue remodeling and fibrosis under inflammatory conditions (15, 16). The distribution of macrophages also changes during development: a transient peak in cochlear macrophage abundance during the second postnatal week coincides with ribbon synapse maturation and is necessary for normal hearing thresholds (17).

**Figure 1:**
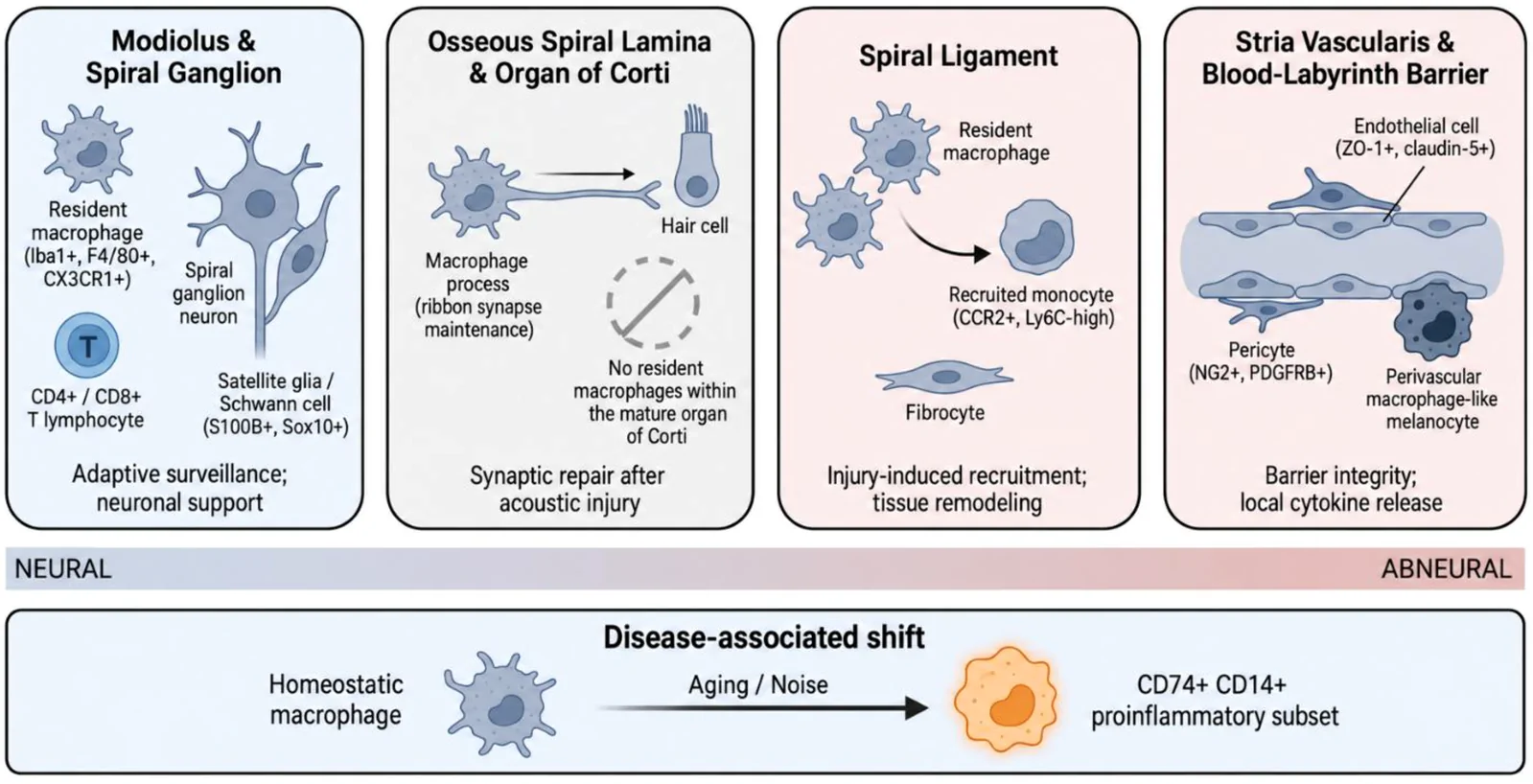
Compartment-resolved cellular composition of the cochlear immune microenvironment. Schematic summary of resident and recruited immune cells across four cochlear compartments, ordered from neural to abneural. The modiolus and spiral ganglion contain resident macrophages, sparse CD4+ and CD8+ T lymphocytes, and satellite glia. Macrophage processes from the osseous spiral lamina reach inner hair cell synapses, whereas the mature organ of Corti lacks resident macrophages. The spiral ligament recruits CCR2+ monocytes after injury. Perivascular macrophage-like melanocytes and pericytes maintain the blood-labyrinth barrier. Aging and noise expand a CD74+CD14+ proinflammatory macrophage subset.

Together, these findings establish the cochlea as an immunologically active organ whose physiology and pathology are shaped by location-specific immune niches, resident macrophage subsets, dynamic BLB function, and crosstalk with the systemic immune system. Understanding SNHL therefore requires placing the immune microenvironment at the center of mechanistic and therapeutic discussions. The resident and recruited immune populations of each major cochlear compartment, together with the associated glial and vascular elements, are summarized by compartment in **Figure 1**.

## 3 Cellular components of the inner ear immune microenvironment

### 3.1 Resident cochlear macrophages

Macrophages are the most abundant immune cell type in the cochlea and constitute the principal effectors of local innate immunity. Under steady-state conditions, resident macrophages occupy distinct niches including the spiral ligament, spiral ganglion, modiolar connective tissue, the stria vascularis as perivascular macrophage-like melanocytes, and the scala tympani surface of the basilar membrane (3). The mature organ of Corti is largely devoid of resident macrophages, although processes from macrophages in the osseous spiral lamina extend beneath inner hair cells and may modulate ribbon synapse function (3, 15). The principal cellular components of the cochlear immune microenvironment, including their anatomical niches, phenotypic markers, and functional roles, are summarized in **Table 1**.

**Table 1:** Cellular components of the cochlear immune microenvironment: anatomical distribution, phenotypic markers, and functional roles.

Cell type/component	Anatomical distribution	Key markers/phenotype	Functional role	References
Yolk sac-derived resident macrophages	Spiral ligament, spiral ganglion, modiolus, scala tympani aspect of basilar membrane	Iba1+, F4/80+, CD68+, CX3CR1+	Local self-renewing; homeostatic surveillance; ribbon synapse maintenance	(3, 12)
Bone marrow-derived monocyte/macrophages	Recruited to spiral ligament and stria vascularis after injury	CCR2+, Ly6Chi (mouse)	Acute infiltration during severe noise, infection, or ototoxicity	(16) (18)
CD74+CD14+ proinflammatory macrophage subset	Expanded near spiral ganglion in ARHL and NIHL	CD74+, CD14+	Driver of inflammation in aging and noise injury; enhanced communication with spiral ganglion neurons	(13)
Perivascular macrophage-like melanocytes (PVM/Ms)	Outside stria vascularis basement membrane	F4/80+, melanin+	Maintain BLB integrity; local cytokine release	(9, 10)
Cochlear nucleus microglia	Central auditory brainstem	Iba1+, CD68+	Activated in aging; C1q deposition; central propagation of cochlear inflammation	(23)
CD4+ T helper lymphocytes	Modiolus, perivascular	CD3+, CD4+	Sparse adaptive surveillance; potential effectors in AIED	(6)
Neutrophils	Infiltrate cochlear tissues during severe inflammation	Ly6G+, MPO+	Acute inflammation; exacerbate NIHL when CX3CR1 signaling is impaired	(21)
Schwann cells and satellite glia	Spiral ganglion; auditory nerve	S100beta+, GFAP+, Sox10+	Neuronal support; myelin maintenance; complement-mediated immune crosstalk	(22)
Cochlear pericytes	Stria vascularis and modiolar capillaries	NG2+, PDGFRbeta+, alpha-SMA+	BLB integrity; vascular control; sensitive to hyperglycemia-induced oxidative stress	(9, 10)
Stria vascularis endothelial cells	Stria vascularis capillaries	CD31+, ZO-1+, claudin-5+	BLB barrier function; primary target of TNF-alpha-mediated permeability increase	(11)

Cochlear macrophages express common myeloid markers including Iba1, F4/80, CD68, and the fractalkine receptor CX3CR1, which can be exploited in transgenic reporter mice to track their dynamics *in vivo* (12, 18). Their origin is mixed: a fraction is derived from yolk sac and self-renews locally, while bone marrow-derived monocytes continuously replenish a portion of the population, with turnover increasing after cochlear injury (3, 18). Single-cell transcriptomic profiling has revealed substantial heterogeneity beyond the conventional M1 and M2 dichotomy. A CD74+CD14+ proinflammatory subset is preferentially expanded in ARHL and NIHL and shows enhanced communication with spiral ganglion neurons through chemokine and cytokine signaling (13). Other studies have identified macrophage subpopulations defined by CX3CR1 and CCR2 expression that differ in their migratory behavior and contributions to injury and repair after noise or infection (16, 18).

The functional outcome of macrophage activity in the cochlea is context-dependent. After acoustic trauma, resident macrophages migrate toward damaged inner hair cell synapses, and pharmacological depletion using the colony-stimulating factor 1 receptor inhibitor PLX5622 reveals that macrophages are required for spontaneous ribbon synapse repair without affecting initial synaptic loss (15). Conversely, lineage-tracing experiments using Ccr2 and Cx3cr1 dual reporters have demonstrated that activated tissue-resident macrophages drive hair cell damage after noise exposure through TLR4 signaling, and that pharmacological TLR4 blockade attenuates this damage (18). These apparently divergent results highlight that the role of macrophages varies according to injury type, severity, time point, and microenvironmental context.

### 3.2 Other immune cell populations

Although less abundant than macrophages, several other immune cell types contribute to cochlear immunity. T lymphocytes, including both CD4+ helper and CD8+ cytotoxic subsets, are detectable in the modiolus and around vessels in human cochleas under steady-state conditions, often in close apposition to macrophages (6). Mast cells, dendritic cells, and B lymphocytes have been described at lower frequencies and may participate in antigen presentation, allergic-type inflammation, or autoantibody responses, especially in autoimmune inner ear disease and Meniere disease (19, 20). Neutrophils are not prominent residents but can infiltrate cochlear tissues during severe inflammatory states, and CX3CR1 signaling appears to restrain their excessive recruitment, as CX3CR1-deficient mice show neutrophil over-infiltration with worsened NIHL (21).

### 3.3 Glia and immune crosstalk

The spiral ganglion is populated by neurons and by satellite glial cells and Schwann cells that ensheath their axons. These glial cells share many features with peripheral nervous system glia and interact intimately with cochlear macrophages. Disruption of complement signaling, for example through loss of complement factor B function, leads to progressive hearing impairment, abnormal auditory nerve myelin, and disrupted gene expression related to neural development and extracellular matrix biology, illustrating the integration between immune signaling and glial neuronal maintenance (22). The aged cochlea additionally shows increased C1q deposition and microglial activation in the central cochlear nucleus, suggesting that peripheral cochlear immune dysregulation propagates centrally and contributes to age-related auditory processing decline (23).

Together, these data establish that the cochlear immune microenvironment comprises resident macrophages of multiple subtypes, recruited monocytes and neutrophils, sparse lymphoid cells, and glial cells embedded in a finely tuned signaling network. The composition and activation state of this network influence the trajectory of every major form of SNHL. As detailed in **Table 1**, this network spans resident and recruited myeloid cells, sparse lymphocytes, glia, pericytes, and stria vascularis endothelium, with each population contributing distinct functions to homeostasis and to disease.

## 4 Molecular drivers of cochlear inflammation

### 4.1 Cytokine and chemokine networks

A consistent feature across cochlear injury models is the rapid upregulation of proinflammatory cytokines, including TNF-alpha, IL-1beta, IL-6, and IL-18, in cochlear tissues following noise, ototoxic, or autoimmune insults (24–26). These mediators are produced by activated macrophages, fibrocytes, and stria vascularis cells, and they amplify the inflammatory response by recruiting additional immune cells and by altering tight junction integrity at the BLB (11). TNF-alpha is particularly potent at disrupting the human BLB endothelial barrier *in vitro* by suppressing tight junction genes and increasing permeability to molecules that are normally excluded (11). The principal molecular mediators of cochlear inflammation discussed in this section, together with their cellular sources, mechanistic roles, and current therapeutic targeting status, are summarized in **Table 2**.

**Table 2:** Key molecular mediators of cochlear inflammation in sensorineural hearing loss: cellular sources, mechanistic roles, and current therapeutic targeting status.

Mediator/pathway	Source in cochlea	Mechanistic role in SNHL	Therapeutic strategy/agent	Development stage	References
TNF-alpha	Macrophages, fibrocytes, stria vascularis cells	BLB endothelial disruption; cytokine amplification; hair cell injury	Etanercept, infliximab	Clinical (AIED, off-label); preclinical in cochlea	(11, 49)
IL-1beta	NLRP3-activated macrophages and hair cells	Pyroptosis; inflammation amplification; central effector in autoinflammatory SNHL	Anakinra, canakinumab	Approved for NLRP3-associated autoinflammatory hearing loss	(35, 50, 51)
IL-6	Cochlear cells, macrophages	Inflammaging; upregulation of Cav1.3 in inner hair cells; synaptic dysfunction	Tocilizumab; IL-6 genetic depletion (research)	Clinical (AIED off-label); preclinical for ARHL	(25)
IL-18	NLRP3 inflammasome-dependent (myeloid cells, hair cells)	Amplifies inflammasome cascade; sustains chronic inflammation	Indirect via NLRP3 inhibition	Preclinical	(26)
HMGB1 (DAMP)	Necrotic and stressed cochlear cells (organ of Corti, lateral wall)	TLR4/RAGE activation; oxidative stress; hair cell loss after noise	Anti-HMGB1 neutralizing antibody; glycyrrhizin	Preclinical	(27, 28)
HSP70/HSP90	Stressed hair cells and supporting cells	DAMP signaling; candidate autoantigen in AIED; perilymph biomarker	Diagnostic and patient-stratification target	Translational/biomarker	(29)
TLR4	Macrophages, supporting cells	DAMP recognition; NF-kappaB activation; cytokine production	TLR4 antagonists; genetic ablation (preclinical)	Preclinical	(18, 24, 30, 31)
Reactive oxygen species/mitochondrial DAMP	Mitochondria; lipid droplets in aged hair cells	Activates NLRP3; drives senescence; central in cisplatin ototoxicity	Sodium thiosulfate; antioxidants	Approved (STS, pediatric cisplatin)/preclinical	(2, 33, 47)

Chemokine signaling determines the spatial and temporal dynamics of immune cell trafficking. The CX3CL1-CX3CR1 axis is central to cochlear macrophage biology. CX3CR1 expression in cochlear macrophages is required for normal ribbon synapse development, and inhibition of CX3CR1 during the critical postnatal window leads to ribbon synapse defects and elevated hearing thresholds (17). In aged stria vascularis, basal cells produce CX3CL1, while macrophages express CX3CR1 and TNF-alpha, and exogenous CX3CL1 enhances macrophage migration and TNF-alpha secretion, identifying the axis as a trigger of cochlear inflammaging (14). CCR2 and other chemokine receptors also influence monocyte recruitment during meningitis-related cochlear injury, and their genetic deletion modifies the severity of hearing loss and ossification (16).

### 4.2 Pattern-recognition receptors and DAMPs

Damage-associated molecular patterns (DAMPs) released from injured cochlear cells serve as endogenous activators of innate immunity. High mobility group box 1 (HMGB1) is a prototypical DAMP whose expression in the cochlea is rapidly upregulated after acoustic injury, particularly in the organ of Corti and lateral wall (27). Neutralization of HMGB1 with antibodies prior to noise exposure attenuates oxidative stress, reduces hair cell loss, and improves auditory brainstem response thresholds in mouse models (28). Heat shock proteins, particularly HSP70 and HSP90, are also released in the inner ear under stress and have been detected in human perilymph, where they correlate with cochlear injury and may serve as biomarkers (29).

Toll-like receptor 4 (TLR4) is a major pattern-recognition receptor on cochlear macrophages and supporting cells. Its activation by DAMPs and by low-molecular-weight hyaluronic acid drives downstream NF-kappaB signaling and amplifies cochlear cytokine production after noise (24, 30, 31). Genetic loss of TLR4 protects against noise-induced sensory cell damage and reduces site-specific inflammation, particularly in the organ of Corti, although effects on the lateral wall are partially preserved, indicating compartment-specific regulation (30). Pharmacological TLR4 inhibition similarly attenuates macrophage-mediated hair cell injury in noise-exposed mice (18).

### 4.3 NLRP3 inflammasome activation and pyroptosis

The NLRP3 inflammasome assembles in response to mitochondrial dysfunction, reactive oxygen species, and DAMP signaling, leading to caspase-1 activation, IL-1beta maturation, and pyroptotic cell death. Multiple lines of evidence implicate NLRP3 in cochlear injury. In NIHL, pharmacological NLRP3 inhibition with agents such as oridonin or selective SETD7 inhibitors reduces hair cell loss and preserves hearing thresholds (26, 32). In senescent cochlear hair cells, lipid droplet accumulation drives NLRP3 activation, and restoration of LXRbeta/ABCA1 signaling attenuates senescence by suppressing the inflammasome (33). Mouse models conditionally expressing mutant Nlrp3 develop progressive hearing loss with cochlear inflammatory change (34), and patients carrying gain-of-function NLRP3 mutations develop autoinflammatory hearing loss that can respond dramatically to IL-1 receptor antagonism, demonstrating the clinical relevance of inflammasome-driven SNHL in humans (35).

### 4.4 Complement signaling

Complement components are expressed in the cochlea and participate in both physiological maintenance and pathological injury. Mice lacking complement factor B develop progressive SNHL, abnormalities of auditory nerve myelin, and stria vascularis alterations, with transcriptomic disturbances in extracellular matrix and neural development pathways (22). In aging cochleas, increased C1q deposition is observed alongside activated microglia in the cochlear nucleus, suggesting that complement activation contributes to both peripheral and central age-related auditory decline (23).

### 4.5 Blood-labyrinth barrier disruption

**Figure 2:**
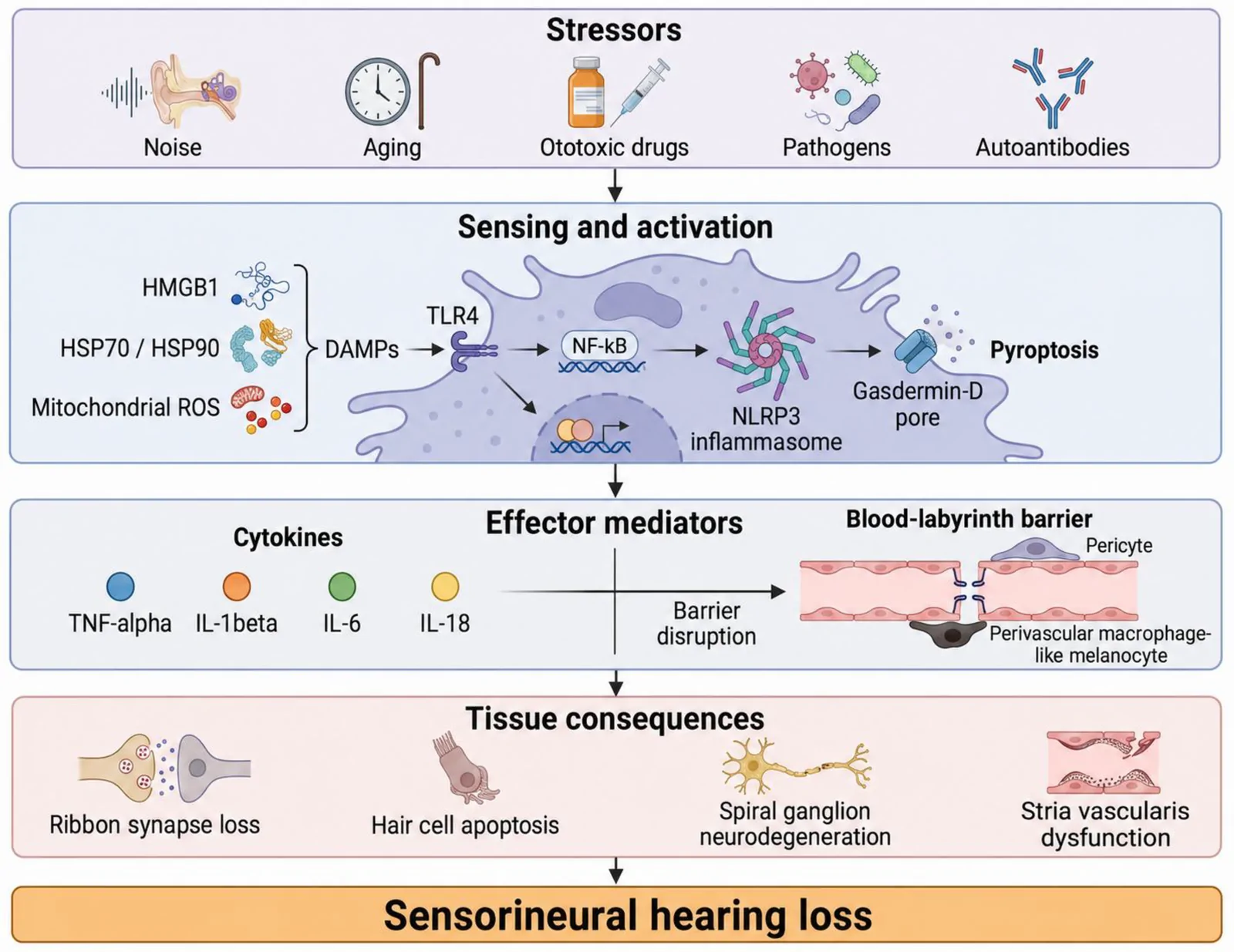
Convergent molecular cascade of cochlear inflammation in sensorineural hearing loss. Diverse stressors converge on a shared inflammatory pathway. Noise, aging, ototoxic drugs, pathogens, and autoantibodies release damage-associated molecular patterns, including HMGB1, HSP70, HSP90, and mitochondrial reactive oxygen species. These engage TLR4 on cochlear macrophages, activating NF-κB and the NLRP3 inflammasome, which drives gasdermin-D pore formation and pyroptosis. Released TNF-α, IL-1β, IL-6, and IL-18 disrupt tight junctions of the blood-labyrinth barrier. The resulting tissue injury converges on sensorineural hearing loss. Complement signaling is discussed in the text.

BLB breakdown is both a consequence of and an amplifier of cochlear inflammation. Cytokines, oxidative stress, and metabolic insults disrupt pericyte-endothelial coupling, reduce tight junction protein expression, and increase paracellular permeability (11). Comparable barrier stress and immune activation follow high-dose cochlear irradiation (36). In diabetic models, hyperglycemia-induced oxidative stress in stria vascularis pericytes triggers mitochondrial apoptosis with subsequent BLB leakage and hearing threshold elevation (37). Restoration of BLB integrity is therefore a potential therapeutic target across multiple SNHL etiologies. The molecular pathways described across this section, from initial stressor exposure through DAMP release, innate immune activation, cytokine production, and barrier disruption, converge on a common inflammatory cascade summarized in **Figure 2**.

## 5 Mechanistic insights across SNHL etiologies

### 5.1 Noise-induced hearing loss

Acoustic overstimulation produces SNHL through mechanical and metabolic stress to hair cells, supporting cells, and synapses, followed by an immune response that can either contribute to repair or worsen damage. Within hours of noise exposure, DAMPs including HMGB1 are released from injured cochlear cells, activating TLR4 and NF-kappaB signaling and inducing TNF-alpha and IL-1beta production in spiral ganglion cells and spiral ligament fibrocytes (24, 27, 28). Low-molecular-weight hyaluronic acid generated during tissue injury further drives TLR4 signaling, and its intracochlear delivery reproduces noise-like inflammation (31).

The role of cochlear macrophages in NIHL is context-dependent. Resident macrophages migrate toward damaged inner hair cell synapses, and *in vivo* macrophage depletion using PLX5622 demonstrates that they are required for spontaneous ribbon synapse repair after moderate noise exposure (15). In contrast, lineage tracing in Ccr2 and Cx3cr1 dual-reporter mice indicates that activated tissue-resident macrophages, rather than infiltrating monocytes, are key drivers of hair cell damage after intense noise, with TLR4 signaling mediating their proinflammatory activation (18). CX3CR1 loss exacerbates NIHL in part by allowing excessive neutrophil infiltration into cochlear tissues (21).

These findings indicate that the cochlear immune response to noise is biphasic and dose-dependent. Moderate insults engage a reparative macrophage response, while severe trauma elicits a damaging proinflammatory response. Therapeutic strategies that selectively target the harmful component, such as TLR4 antagonism, NLRP3 inhibition, or HMGB1 neutralization, have shown protective effects in preclinical noise models (26, 28, 30, 32). The clinical translation of these strategies for occupational, military, or recreational noise exposure remains an active area of investigation.

### 5.2 Age-related hearing loss

ARHL is the most common form of SNHL and is increasingly conceptualized as a disease of cochlear inflammaging. Inflammaging refers to the chronic, low-grade, sterile inflammation that accompanies aging and contributes to numerous age-related disorders (4, 38). In the cochlea, aging is associated with persistent elevation of TNF-alpha, IL-6, and IL-1beta, increased macrophage and microglial activation, and progressive stria vascularis dysfunction with BLB compromise (10, 23, 38).

Macrophage dynamics are altered in the aged cochlea. Single-cell transcriptomic studies show expansion of the CD74+CD14+ proinflammatory macrophage subset with enhanced communication with spiral ganglion neurons, paralleling the loss of these neurons in ARHL (13). In the aged stria vascularis, basal cell CX3CL1 and macrophage CX3CR1/TNF-alpha expression are increased, and exogenous CX3CL1 amplifies macrophage migration and TNF-alpha secretion, suggesting that the fractalkine axis is a trigger for local inflammatory remodeling (14). IL-6 produced in the cochlea further drives functional changes in inner hair cells by upregulating Cav1.3 calcium channels via JAK-MAPK signaling, leading to neurotransmitter excitotoxicity and synaptic impairment that can be attenuated by IL-6 deficiency or calcium channel blockade (25).

Inflammaging interacts with established ARHL mechanisms, including hair cell senescence, mitochondrial DNA damage, and reduced antioxidant capacity. Lipid droplet accumulation in aging hair cells activates the NLRP3 inflammasome, and restoration of LXRbeta/ABCA1 signaling reduces senescence in part by suppressing the inflammasome (33). Vestibular end-organs share many of these inflammatory and oxidative mechanisms, suggesting common substrates for age-related cochlear and vestibular decline (39). Importantly, cochlear inflammaging propagates centrally, with C1q deposition and microglial activation in the cochlear nucleus paralleling peripheral changes (23).

These mechanistic insights provide a rationale for targeting cochlear inflammaging as a strategy to prevent or slow ARHL. Candidate approaches include senolytics, NLRP3 inhibitors, IL-6 axis modulators, antioxidants combined with anti-inflammatory agents, and stria vascularis-directed interventions to preserve BLB function and the endocochlear potential (10, 40).

### 5.3 Ototoxic sensorineural hearing loss

Cisplatin and aminoglycoside antibiotics are major causes of preventable SNHL, with cisplatin alone responsible for hearing loss in approximately 40 to 60 percent of treated adults and up to 60 percent of treated children (41, 42). Cisplatin enters cochlear hair cells through copper transporters and TRPV1 channels, generating reactive oxygen species, mitochondrial dysfunction, and DNA damage that initiate intrinsic apoptosis, necroptosis, pyroptosis, and ferroptosis (43–45). Critically, cisplatin also activates a robust cochlear inflammatory response, with macrophage infiltration and proinflammatory cytokine production amplifying hair cell loss and contributing to spiral ganglion injury (42, 43).

Aminoglycoside ototoxicity involves TRPV1-mediated drug uptake into hair cells, mitochondrial damage, and JNK signaling, with CDK2 regulating c-Jun activation downstream (45, 46). As with cisplatin, secondary inflammation appears to amplify direct cellular toxicity. Sodium thiosulfate is now approved in pediatric oncology to reduce cisplatin-induced hearing loss, acting by direct platinum inactivation and intracellular antioxidant effects, although its compatibility with antitumor efficacy continues to be debated (47). Anti-inflammatory adjuncts, including TRPV1 modulators, NLRP3 inhibitors, antioxidants, and intratympanic delivery systems, are under active development to complement otoprotectants and reduce ototoxic SNHL (42–44).

### 5.4 Autoimmune and autoinflammatory inner ear disease

Autoimmune inner ear disease (AIED) is a rare but instructive form of immune-mediated SNHL, presenting as bilateral, asymmetric, rapidly progressive hearing loss often responsive to corticosteroids (48). Although a single pathognomonic biomarker has not been identified, autoantibodies against inner ear antigens including HSP70, T-cell and B-cell infiltration in animal models, and association with systemic autoimmune disorders provide collective evidence for an autoimmune pathogenesis (48). Cogan syndrome exemplifies a systemic vasculitis that targets the inner ear and eye and can cause irreversible deafness if untreated, with emerging use of biologic agents in steroid-refractory cases (49).

Autoinflammatory conditions provide direct human evidence for inflammasome-driven SNHL. Patients with Muckle-Wells syndrome and other NLRP3-associated autoinflammatory diseases develop progressive SNHL that can be stabilized or even reversed with the IL-1 receptor antagonist anakinra (35, 50, 51). Mouse models with conditional expression of mutant Nlrp3 in the cochlea recapitulate severe hearing loss with characteristic histopathology, confirming the cochlea as a direct target of inflammasome dysregulation (34).

Meniere disease, characterized by episodic vertigo, fluctuating SNHL, and tinnitus, also shows features consistent with a cochlear inflammatory disorder. Inner ear cytokines including IL-1beta and TNF-alpha are elevated, and a subset of patients exhibits autoimmune comorbidities or allergic phenotypes detectable on systemic immune profiling, suggesting opportunities for personalized immunomodulation (19, 20, 52).

### 5.5 Sudden sensorineural hearing loss and viral SNHL

Sudden sensorineural hearing loss (SSNHL) is defined as a hearing decrease of at least 30 dB across three contiguous frequencies developing within 72 hours and affects approximately 5 to 27 per 100,000 people annually (8). Most cases are idiopathic, but proposed mechanisms include cochlear ischemia, viral infection, autoimmune injury, and acute cochlear inflammation (53). The standard of care includes systemic and intratympanic corticosteroids, which provide modest benefit and presumably act by suppressing cochlear inflammation, although treatment response is highly variable (7, 8).

Viral and post-viral SSNHL has gained renewed attention after the COVID-19 pandemic. SARS-CoV-2 infection has been associated with SSNHL in case series and systematic reviews, with proposed mechanisms including direct viral cytopathy, cytokine storm, and microvascular thrombosis (54, 55). Population-based cohort data have not consistently confirmed increased SSNHL incidence after COVID-19 vaccination, but inflammation-driven cochlear injury remains plausible in selected patients (56).

### 5.6 Radiation-related sensorineural hearing loss

Therapeutic head and neck radiation can cause acute and delayed SNHL. In mouse models, high-dose cranial irradiation induces SNHL within days, accompanied by spiral ganglion neuron atrophy and reduced neurofilament expression, while hair cell loss is minimal at early time points (36). The cochlear immune microenvironment is reshaped after irradiation, with transient upregulation of inflammatory cytokines and progressive reduction in resident macrophage numbers over days to weeks (36). These observations argue that radiation-induced SNHL is not solely a hair cell phenomenon but reflects a complex interaction among neural injury, immune dysregulation, and BLB disruption, with implications for otoprotective strategies in head and neck oncology.

## 6 Therapeutic perspectives

### 6.1 Corticosteroids and the rationale for immunomodulation

Systemic and intratympanic corticosteroids remain the principal evidence-based therapy for SSNHL and AIED, with their efficacy attributed to broad suppression of cochlear inflammation, edema, and oxidative stress (8). Cochrane analyses indicate that intratympanic corticosteroids provide modest benefit when used as primary therapy and somewhat more substantial benefit as salvage therapy after failure of systemic treatment, although the certainty of evidence remains low (7). Adjunctive hyperbaric oxygen therapy may provide additional gains in selected patients, particularly when initiated early (57). Despite their persistence as the standard of care, corticosteroids are nonspecific and carry systemic and local side effects, motivating the development of targeted alternatives.

### 6.2 Cytokine-directed biologics

Biologic agents that selectively neutralize cytokines or block their receptors have transformed the treatment of systemic immune-mediated diseases and offer rational candidates for immune-mediated SNHL. The strongest clinical evidence is for IL-1 inhibition in NLRP3-associated autoinflammatory disorders, in which anakinra can stabilize or even reverse progressive SNHL, including in pediatric Muckle-Wells syndrome (35, 50, 51). TNF-alpha blockade with etanercept or infliximab has been used in selected steroid-refractory AIED cases, while IL-6 inhibition with tocilizumab has emerged as a promising option for steroid-resistant disease and is supported by preclinical data implicating IL-6 in inflammaging-related ARHL (25, 49). Personalized immune profiling in Meniere disease has identified subsets that may benefit from IL-4 or IgE-directed strategies, illustrating the broader move toward biomarker-stratified biologic therapy (19, 20).

### 6.3 Inflammasome and pyroptosis inhibitors

The NLRP3 inflammasome is a particularly attractive target because of its central position in the convergence of oxidative, metabolic, and DAMP signals. Preclinical studies have demonstrated otoprotection with natural-product NLRP3 inhibitors such as oridonin, with SETD7 inhibitors such as (R)-PFI-2 that suppress NF-kappaB and NLRP3 assembly, and with autophagy enhancers such as tranylcypromine that indirectly restrict inflammasome activation (26, 32, 58). Genetic and pharmacological data in autoinflammatory disease provide proof of concept that inflammasome blockade can preserve human hearing (34) (35).

### 6.4 Complement-targeted therapy

Loss-of-function studies indicate that complement components are essential for normal auditory function, while excessive complement activation is observed in aging cochleas and may contribute to neuronal and stria vascularis injury (22, 23). Complement-targeted therapies such as C1 inhibitors and factor B and D inhibitors, which are advancing in age-related macular degeneration and other indications, could be repurposed for cochlear disorders, although their narrow therapeutic windows demand careful preclinical validation.

### 6.5 Macrophage modulation

Given the dual protective and pathogenic roles of cochlear macrophages, modulation rather than depletion is the more rational strategy. Pharmacological tools that influence macrophage polarization, including CSF1R modulators and TLR4 antagonists, have been used preclinically to dissect macrophage contributions to NIHL (15, 18). Selective targeting of pathogenic macrophage subsets, such as the CD74+CD14+ population identified in ARHL and NIHL, may provide further specificity in future therapeutic design (13).

### 6.6 Cell and vesicle-based therapy

Mesenchymal stem cell (MSC)-derived exosomes are emerging as a low-immunogenicity, cell-free strategy with anti-inflammatory and pro-regenerative effects. Umbilical cord MSC exosomes administered systemically or via the round window niche reduce hair cell loss and preserve hearing in cisplatin-injected mice and produce transcriptomic changes consistent with tissue remodeling (59). MSC-derived exosomes also protect against neomycin-induced hair cell damage *in vivo*, in part by activating autophagy (60). Engineered exosomes derived from neural progenitor cells overexpressing miR-21 reduce inflammatory cytokines and improve auditory thresholds in cochlear ischemia-reperfusion models, illustrating how exosome cargo can be tailored to specific molecular targets (61).

### 6.7 Local delivery innovations

Effective local delivery is critical because systemic drug penetration into the cochlea is limited by the BLB. Intratympanic injection of corticosteroids is established clinically, but newer approaches aim to improve sustained release and tissue specificity. Poly(lactic-co-glycolic acid) nanoparticles loaded with dexamethasone and administered via a mastoid approach yield higher perilymph concentrations than intraperitoneal or intratympanic routes and confer better protection against lipopolysaccharide- and noise-induced damage (62). Crosslinked hybrid nanoparticles embedded in thermoresponsive hydrogels enable co-delivery of multiple otoprotective molecules with sustained release for at least one month, providing a platform for combination therapy against ototoxic SNHL (63).

### 6.8 Gene therapy and the immune microenvironment

Adeno-associated virus (AAV)-based gene therapies are advancing rapidly for monogenic SNHL, with successful preclinical and early clinical outcomes for otoferlin-related deafness, Usher syndrome type 1F, and DFNB1 caused by Gjb2 mutations (64–66). Although these strategies primarily target hair cell function rather than immunity, they have important implications for the cochlear immune microenvironment. AAV delivery must be designed to minimize ectopic expression and host immune responses, with cell-type-specific promoters and serotype selection now enabling tightly regulated expression in targeted cochlear cells (64, 66). The same delivery platforms could ultimately be repurposed to express immunoregulatory molecules locally, providing a route to long-term modulation of cochlear inflammation.

### 6.9 Perilymph liquid biopsy and precision immunotherapy

A major advance in human cochlear research has been the development of safe perilymph sampling techniques during cochlear implantation, enabling characterization of the *in vivo* cochlear microenvironment without compromising residual hearing (29). Systematic analyses of perilymph from patients with SNHL have identified candidate biomarkers grouped into heat shock proteins, complement components, microRNAs, and neurotrophic and structural proteins, with HSP70, HSP90, complement components, miR-1299, miR-1270, and brain-derived neurotrophic factor-regulated proteins emerging as priority targets for validation (29). Integrating perilymph multi-omics with audiometric phenotyping could enable mechanism-based stratification of patients for tailored anti-inflammatory, biologic, or gene-based therapies in coming years.

## 7 Knowledge gaps and future directions

Despite substantial progress, important gaps remain in understanding the inner ear immune microenvironment and translating that understanding to clinical practice. Most mechanistic data derive from rodent models, and species differences in cochlear immune cell composition, gene expression, and pharmacology limit direct extrapolation to humans. Comprehensive single-cell and spatial transcriptomic atlases of the healthy and diseased human cochlea, drawn from temporal bone donors and from perilymph sampling, are needed to verify which findings translate (6, 13, 29).

The functional duality of cochlear macrophages, in which the same cell type can either repair synapses or drive sensory cell damage depending on context, requires more refined dissection. Subset-specific markers, lineage-tracing strategies, and conditional genetic tools will be essential to define which macrophage populations to spare and which to target therapeutically (13, 15, 18). Similarly, the contribution of T cells, B cells, and innate-like lymphocytes to cochlear pathology remains underexplored, particularly in AIED and Meniere disease, where targeted lymphocyte modulation may be more appropriate than nonspecific immunosuppression (19, 20).

Sex differences in cochlear immunity and SNHL epidemiology are recognized but mechanistically unexplained, with men generally showing higher prevalence and greater noise-induced damage (1). Whether these differences reflect cochlear immune cell composition, hormonal influences, or systemic immune phenotypes is unknown and represents a major opportunity for future research.

Clinical trial design for immune-targeted cochlear therapies poses unique challenges, including the placebo response in SSNHL, the difficulty of blinding intratympanic interventions, the lack of validated mechanistic biomarkers, and the heterogeneity of patient populations grouped under broad clinical labels such as idiopathic SNHL (7, 8). Perilymph biomarker discovery, advanced cochlear imaging, and biomarker-guided stratification will be essential to move beyond underpowered, indication-broad trials.

Finally, the cochlear immune microenvironment intersects with central auditory pathways and with systemic inflammatory states such as inflammaging, metabolic syndrome, and chronic infections (23, 38, 53). Future strategies are likely to integrate local cochlear interventions with systemic risk factor modification, exemplified by trials targeting cardiovascular health, metabolic control, and infection prevention as components of comprehensive hearing preservation. Bridging otology, immunology, computational biology, and population health will be essential to realize the full clinical potential of this rapidly evolving field.

## 8 Conclusion

The inner ear immune microenvironment has emerged as a central determinant of sensorineural hearing loss across diverse clinical etiologies, from noise and aging to ototoxicity, autoimmune disease, sudden deafness, viral injury, and radiation. Resident macrophages, lymphocytes, glia, and the BLB together form a dynamic network whose activation patterns shape both the pathogenesis and the reparative potential of cochlear injury. Convergent molecular drivers, including cytokines, DAMPs, NLRP3 inflammasome activation, and complement signaling, provide common therapeutic entry points across otherwise distinct conditions. Translation of these mechanistic insights into precision immunomodulation, supported by perilymph biomarker discovery and advanced drug delivery, holds the promise of moving SNHL therapy beyond broad corticosteroid use toward biomarker-guided, mechanism-specific interventions. Realizing this vision will require sustained interdisciplinary collaboration between otology, immunology, and computational biology, with the ultimate goal of preserving hearing and quality of life across the lifespan.
